# Supplementary material for: Rad52-Rad51 association is essential to protect Rad51 filaments against Srs2, but facultative for filament formation
Source: eLife. 2018 Jul 9;7:e32744. doi: 10.7554/eLife.32744 (PMC6056232; doi:10.7554/eLife.32744)
Supplement: Supplementary file 1. [file elife-32744-supp1.docx]

**Supplementary file 1**

|  | **Nucleotide change** | **Triplet Change** | **Amino acid change** | **Plasmid number** |
| --- | --- | --- | --- | --- |
| **Random mutagenesis** | T1124C | GTC->GCC | V375A | pEc78 |
|  | T1135A | TTT->ATT | F379I | pEc68 |
|  | G1138A | GCA->ACA | A380T | pEc66 |
|  | C1141T | CCA->TCA | P381S | pEc65, pEc75* |
|  | C1142T | CCA->CTA | P381L | pEc67 |
|  | G1167T | AAG->AAT | K389N | pEc76 |
| **Directed mutagenesis** | G1111A | GCG->ACG | A371T | pEc105 |
|  | T1120G | TCT->GCT | S374A | pEc106 |
|  | T1126G | TAT->GCT | Y376A | pEc107 |
|  | AA1132-33GC | AAA->GCA | K378A | pEc108 |
|  | G1148C | GGA->GCA | G383A | pEc109 |
|  | AG1159-60GC | AGC->GCC | S387A | pEc110 |

* Selected twice
