# Supplementary material for: Rad52-Rad51 association is essential to protect Rad51 filaments against Srs2, but facultative for filament formation
Source: eLife. 2018 Jul 9;7:e32744. doi: 10.7554/eLife.32744 (PMC6056232; doi:10.7554/eLife.32744)
Supplement: Supplementary file 2. [file elife-32744-supp2.docx]

**Supplementary file 2. *S. cerevisiae* strains.**

| Strain | Genotype | Source | Strain background* |
| --- | --- | --- | --- |
| FF18733 | *MAT***a** *leu2-3, 112 trp1-289 ura3-52 lys1-1 his7-2* | F. Fabre |  |
| FF18742 | *MAT***a** *rad52::URA3* | F. Fabre |  |
| ECS2136 | *MAT***a** *rad52::URA3 srs2::NAT* |  |  |
| FF18964 | *MAT***a** *rad50::URA3* | F. Fabre |  |
| FF18973 | *MAT***a** *rad54::LEU2* | F. Fabre |  |
| FF181495 | *MAT***a** *sgs1::URA3* | F. Fabre |  |
| ECS57 | *MAT***a** *rrm3::NATMX* |  |  |
| ECS74 | *MAT***a** *mrc1::NATMX* |  |  |
| ECS75 | *MAT***a** *ctf18::NATMX* |  |  |
|  |  |  |  |
| ECS2928 | *MAT***a** *rad51::URA3 YCplcac111-RAD52-6H-FFF* |  |  |
| ECS2947 | *MAT***a** *rad52::URA3 YCplcac111-RAD52-6H-FFF* |  |  |
| ECS2948 | *MAT***a** *rad51::URA3 YCplcac111-rad52-YEKF∆-6H-FFF* |  |  |
| ECS2987 | *MAT***a** *rad51::URA3 YCplcac111-rad52-S374A-6H-FFF* |  |  |
| ECS2929 | *MAT***a** *rad51::URA3 YCplcac111-rad52-Y376A-6H-FFF* |  |  |
| ECS2930 | *MAT***a** *rad51::URA3 YCplcac111-rad52-K378A-6H-FFF* |  |  |
| ECS2931 | *MAT***a** *rad51::URA3 YCplcac111-rad52-P381S-6H-FFF* |  |  |
| ECS2932 | *MAT***a** *rad51::URA3 YCplcac111-rad52-G383A-6H-FFF* |  |  |
| ECS2917 | *MAT***a** *rad51::URA3 YCplcac111* |  |  |
|  |  |  |  |
| L317.4a | *MAT***a** *arg4-RV* |  |  |
| L318.1d | *MAT*α *arg4-Bg* |  |  |
| L317.17c | *MAT***a** *arg4-RV srs2::LEU2* |  |  |
| L318.7a | *MAT*α *arg4-Bg srs2::LEU2* |  |  |
| L317.10d | *MAT***a** *arg4-RV rad52-P381S* |  |  |
| L318.6D | *MAT*α *arg4-Bg rad52-P381S* |  |  |
| L317.3a | *MAT***a** *arg4-RV rad52-P381S srs2::LEU2* |  |  |
| L318.1c | *MAT*α *arg4-Bg rad52-P381S srs2::LEU2* |  |  |
| L340.12c | *MAT***a** *arg4-RV rad52-Y376A* |  |  |
| L342.7a | *MAT*α *arg4-Bg rad52-Y376A* |  |  |
| L340.3b | *MAT***a** *arg4-RV rad52-Y376A srs2::LEU2* |  |  |
| L342.1b | *MAT*α *arg4-Bg rad52-Y376A srs2::LEU2* |  |  |
|  |  |  |  |
| EMY110 | *ade1-100 ura3-52 leu2-3,112 lys5 hml::ADE1 mat::hisG hmr::ADE1 leu2-cs his4::NAT-leu2∆5’ ade3::GAL::HO RAD52-flag::KANMX* | J. Haber | YFP17 |
| EMY108 | *srs2::KanMX* |  | YFP17 |
| EMY379 | *rad52-P381S* |  | YFP17 |
| EMY373 | *rad52-P381S srs2::LEU2* |  | YFP17 |
| ECS3220 | *rad52-Y376A* |  | YFP17 |
| ECS3222 | *rad52-Y376A srs2::LEU2* |  | YFP17 |
|  |  |  |  |
| tGI354 | *hml::ADE1 MATalpha hmr::ADE1 arg5,6::MATa-inc::HPH1 ade3::GAL::HO* | J. Haber | *JKM146* |
| ECS2632 | *srs2::KanMX* |  | *JKM146* |
| EMY402 | *rad52-P381S* |  | *JKM146* |
| EMY352 | *rad52-P381S srs2::LEU2* |  | *JKM146* |
| TLM208 | *rad52-Y376A* |  | *JKM146* |
| TLM209 | *rad52-Y376A srs2::LEU2* |  | *JKM146* |

* When different from FF18733.
